# Antioxidant activities of two sweet pepper *Capsicum annuum *L. varieties phenolic extracts and the effects of thermal treatment

**Published:** 2013

**Authors:** Narmin Yazdizadeh Shotorbani, Rashid Jamei, Reza Heidari

**Affiliations:** 1 *Department of Biology, Faculty of Science, Urmia University, Urmia, I. R. Iran*

**Keywords:** Antioxidant activity, * Capsicum annuum *L., Phenolic extracts, Temperature

## Abstract

**Objectives:** Sweet peppers *Capsicum annuum* L. (*C. annuum*) are an excellent source of vitamins A and C as well as phenolic compounds, which are important antioxidant components that may reduce the risk of diseases. The objective of this study was to evaluate their antioxidant activity under various temperatures.

**Materials and Methods: **To compare the antioxidant activity in various temperatures (20, 35, 50, and 65 °C), two different types of colored (red and green) sweet bell peppers *C annuum* were selected. The red peppers were selected from those cultivated in Shahreza, Esfahan and the green peppers with the local name of Gijlar were selected from those cultivated in Urmia, West Azarbayjan. The experiments were carried out to measure the total phenolic and flavonoid content, ferric reducing antioxidant power (FRAP), chain-breaking activity, scavenging activities of 2,2-diphenyl-1-picrylhydrazyl (DPPH), and hydrogen peroxide radicals.

**Results:** Total phenol and flavonoid contents of pepper extracts were enhanced with increasing temperature to 65 °C. Scavenging capacity of DPPH radical of red pepper extract was enhanced because of putting at 50 °C for 30 min and for Gijlar pepper extract scavenging capacity was increased at 65 °C. Scavenging capacity of hydrogen peroxide radical of extracts was the highest at 35 °C. Chain-breaking activity of red pepper extract was increased for 60 min at 35 °C. FRAP (C) of red pepper extract was significantly different (p<0.05) in compare with Gijlar pepper.

**Conclusion:** An appropriate temperature maintained a high antioxidant activity of phenolic compound, which could be due to the combined effect of non enzymatic reaction and phenolic compound stability.

## Introduction

Bell peppers, also commonly referred to as capsicum, have been known for their antioxidant properties. Available in different colors (green, yellow, orange, and red), bell pepper is studied widely for its beneficial effects in different disorders; especially in disorders related to mental health. The bell pepper contains different bioactive compounds along with significant amounts of beta-carotene (pro vitamin A) and other similar compounds. When compared with the color of bell pepper and the levels of active contents, red bell pepper had the highest amount of beta-carotene and yellow bell pepper had the lowest levels. All bell peppers were noted to have equivalent amounts of antioxidant activity. When used for cooking, it was noted that bell pepper prevented the oxidation of essential fatty acids (Sun et al., 2007 ▶). A study performed on rats established the antioxidant ability of bell pepper which has protective effects on the brain cells. The chemical compounds present in bell pepper actively prevented oxidation of the essential fats within the brain cells that are considered necessary for optimal brain function (Oboh and Rocha, 2008 ▶). Antioxidants are micronutrients that have gained importance in recent years due to their ability to neutralize free radicals or their actions (Cadenas and Packer, 1996 ▶). Free radicals have been implicated in the etiology of several major human ailments, including cancer, cardiovascular diseases, neural disorders, diabetes, and arthritis (Sies, 1996 ▶). In recent years, peppers have grown in popularity and a wide number of varieties are now available in the grocery stores. Almost all peppers turn from green to yellow, orange, red, or purple when they are fully ripe. Green bell peppers are often harvested before they are ripe, and changes in the maturity may affect the content of phytonutrients, which play an important role in the diet antioxidant intake. Fresh pepper is one of the vegetables that has a higher content of vitamin C (Vanderslice et al., 1990 ▶). Phenol compounds show good antioxidant ability (Duan et al., 2007 ▶), but they are relatively unstable (Zhang et al., 2000 ▶). The stability of phenol compounds is dependent on various factors, such as pH value and temperature (Zhang et al., 2001 ▶).The objective of this study was to measure the total phenolic content, flavonoid content, FRAP, chain-breaking activity, scavenging activities of DPPH, and hydrogen peroxide radicals under various temperatures.

## Materials and Methods

**Plant materials**

The samples from two different color (red and green) sweet bell peppers *C. annuum* variety were respectively obtained from agriculture research centers in Esfahan and West Azarbayjan in March and July 2011 and were recognized by these centers.

**Extraction**

One fresh pepper was cut in half and the inside seeds were removed. Then each pepper was separately cut into small pieces and grounded using a kitchen blender. Each of the pepper slurries (200 g) was added to a glass beaker and homogenized with 200 ml of methanol. The mixture was incubated at 45 °C in the water bath with gentle stirring for 30 min. The homogenate was filtered through Whatman No. 1 filter paper to obtain a clear supernatant. The supernatant was transferred to a clean flask AND the residue was mixed with another 100 ml of methanol to repeat the extraction. The resulting supernatant was combined with the previous one. The methanol in the supernatant evaporated under vacuum at 45 °C using a vacuum centrifuge evaporator and the pepper extract reduced to 100 ml. The solution was sealed and stored at 4 °C until use (Sun et al., 2007 ▶).

**Determination of total phenol content**

Total phenolic content of the extracts was determined using the Folin-Ciocalteau reagent (Horwits, 1984). Folin-Ciocalteau reagent was diluted 10 times with distilled water. The pepper extract solution (20 μL) was mixed with 1 ml diluted Folin-Ciocalteau reagent, 1 ml sodium bicarbonate solution (7.5%), and 1 ml distilled water. The mixture was incubated at room temperature for 15 min. The absorbance of the solution was determined at 730 nm using a spectrophotometer (Biowave, S2100, UK) and compared with agallic acid equivalents (GAE) calibration curve. The total phenolic content was expressed as mg gallic acid equivalents of 100 gram fresh pepper.

**Determination of total flavonoid content**

Flavonoid content was determined, as described by Bonvehi et al. (2001) ▶, with some modifications. An appropriate dilution (0.2 ml extract, 0.8 ml distilled water) of the extract was mixed with the same volume (1 ml) of 2% AlCl_3_ in methanol solution (5% acetic acid in methanol). The mixture was allowed to react for 10 min and the absorbance was read at 430 nm against a sample blank without reactants. Quercetine was used as standard for the calibration curve. Total flavonoid content of the extracts was expressed as mg quercetine equivalents (QE) of 100 gram fresh pepper.

**Determination of DPPH radical scavenging activity**

The free radical scavenging capacity of extracts was determined using DPPH )Burits and Bucar, 2000 ▶). Two ml of freshly prepared methanol solution of DPPH (0.004%) was added to 20 μL of extracts and allowed to stand at room temperature for 30 min. The absorbance (A) of sample solution was measured at 517 nm, compared with that of control solution (maximum absorbance). Control solution was prepared containing the same volume without any extract. Scavenging percentage of the DPPH free radical was measured using the following equation: DPPH radical scavenging percentage= [(A_Control _–A_Sample_)/A_Control_] ×100

**Chain-breaking activity assessment**

The chain-breaking activity was based on the method of Brand-Williams et al. (1995) ▶. In order to determine this activity, a volume of 1.9 ml of 6×10^-5^ M DPPH in methanol was added to 10 μL of extracts. After 60 min incubation at room temperature, absorbance was read at 515 nm using a spectrophotometer. The chain-breaking activity was expressed by the reaction rate k and calculated by the following equation: Abs^-3^-Abs_0_^-3^=-3kt

Where Abs_0_ is initial absorbance, Abs is absorbance at increasing time, (t), and the reaction rate was expressed as k. Antioxidant activity was reported as (-Abs^-3^/min/mg FW).

**Hydrogen peroxide radical inhibition assay**

A modified version of the method described by Ruch et al. (1989) ▶ was used to determine the hydrogen peroxide scavenging ability of extracts. Ten μL of extracts was dissolved in 3.4 ml of a 0.1 M phosphate buffer (pH 7.4) solution and mixed with 600 μL of a 43 mM solution of hydrogen peroxide (prepared in the same buffer). The absorbance of these solutions was measured at 230 nm against the corresponding blank solutions. Hydrogen peroxide scavenging capacities of the extracts were calculated using the following equation: Scavenging percentage = [(A_Blank_ – A_Sample_)/A_Blank_] × 100.

Where A_Sample_=absorbance of reaction mixture and A_Blank_=absorbance of blank mixture (distilled water instead extract).

**Reducing power using** **FRAP assay**

The reducing power was determined using FRAP assay described by Benzie and Strain (1996) ▶ with some modifications. Briefly, the FRAP reagent contained 2.5 ml of 10 mM 2,4,6-trypyridyl-s-triazine (TPTZ) solution in 40 mM HCl plus 2.5 ml of 20 mM FeCl_3_ and 25 ml of 0.3 M acetate buffer (pH 3.6) were freshly prepared. Thirty μL of extract was mixed with 3 ml of FRAP reagent and the absorption of the reaction mixture was measured at 595 nm. Methanolic solutions of known Fe (II) concentration, in the range of 60-1000 mmol/L (FeSO_4_), were used for obtaining the calibration curve. The FRAP value represents the ratio between the slope of the linear plot for reducing Fe^3+^-TPTZ reagent by pepper extract compared with the slope of the plot for FeSO_4_.

**Statistical analysis**

All the assays were carried out in triplicate. The results are expressed as mean values and standard error (SE) of the mean. The differences were analyzed using one-way analysis of variance (ANOVA) followed by Tukey's tests. For all analyses, p-values<0.05 were considered statistically significant. Data were analyzed using SPSS version 16 program.

## Results

**Figure 1:**
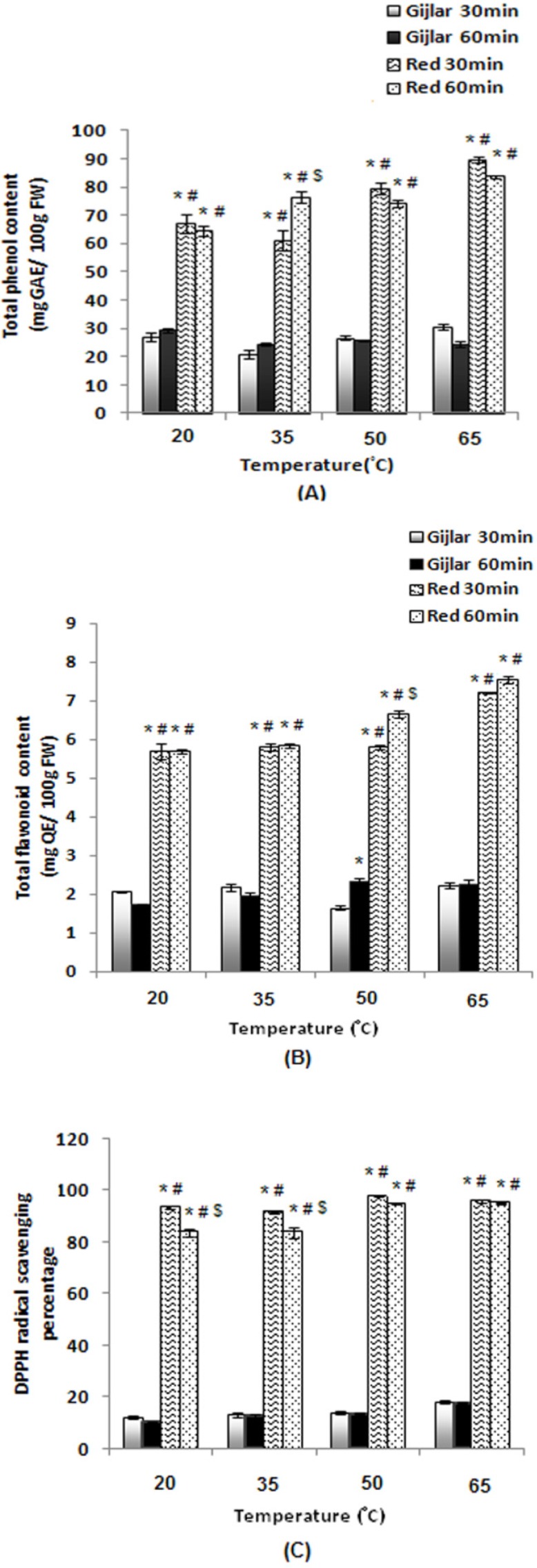
The effects of various temperatures on total phenol content (A), total flavonoid content (B), and scavenging activity of DPPH radical (C) of extracts. Data are represented as mean values and standard error (SE) of the mean. Statistical comparisons were carried out by one way ANOVA followed by Tukey´s tests. Vertical bars represent the SE. *p<0.05 Gijlar 30min compared to another groups, #p<0.05 Gijlar 60min compared to another groups, $p<0.05 Red 30min compared to Red 60min

**Figure 2:**
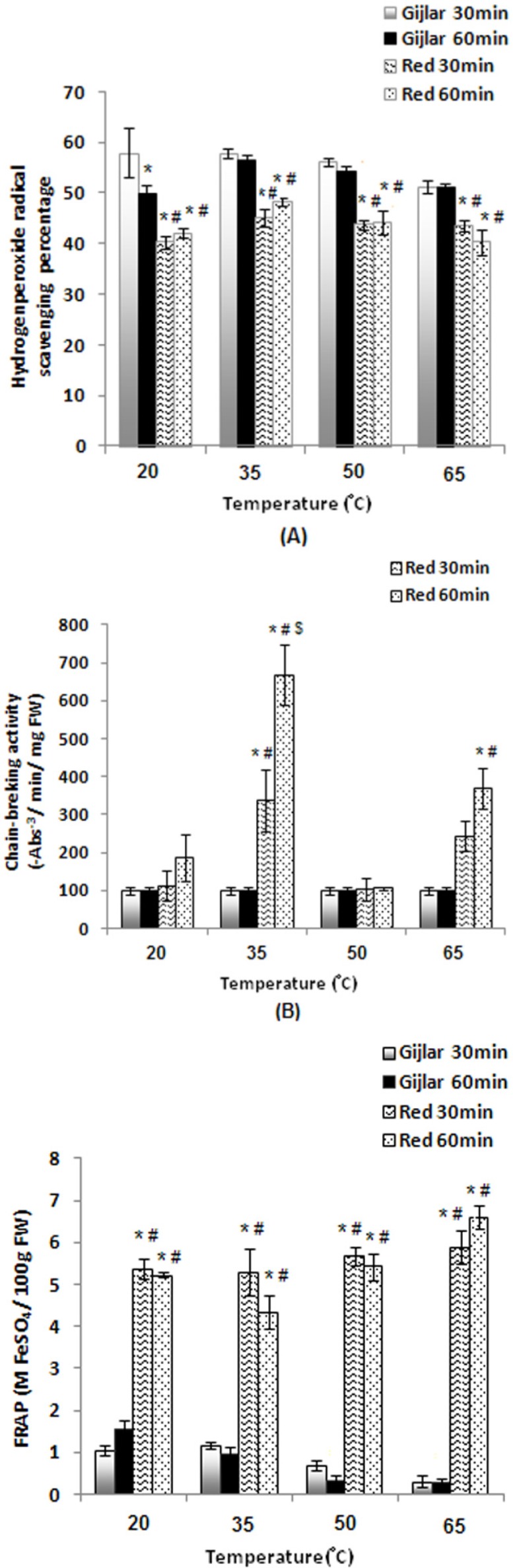
The effects of various temperatures on scavenging activity of hydrogen peroxide radical (A), chain-breaking activity (B), and FRAP (C) of extracts. Data are represented as mean values and standard error (SE) of the mean. Statistical comparisons were carried out by one way ANOVA followed by Tukey´s tests. Vertical bars represent the SE. *p<0.05 Gijlar 30min compared to another groups, #p<0.05 Gijlar 60min compared to another groups, $p<0.05 Red 30min compared to Red 60min

The antioxidant activities in extracts of green and red peppers were determined within various temperatures. As shown in Figure 1, total phenol content (A) of extracts was enhanced with increasing temperature to 65 °C, but it decreased as the incubation time was extended to 60 min. Furthermore, total phenol content in red peppers was high and significantly different (p<0.05) in compare with Gijlar pepper. Total flavonoid content (B) of extracts was enhanced with increasing temperature to 65 °C, and it increased as the incubation time was extended to 60 min, which suggested that pepper phenolics are relatively stable under high temperature conditions. Total flavonoid content of Gijlar pepper for 30 min at 50 °C was the lowest level and for 60 min at this temperature was the highest level. Total flavonoid content of red pepper was significantly different (p<0.05) in compare with Gijlar pepper. Scavenging activity of DPPH radical (C) of red pepper extract for 30 min at 50 °C was increased. Scavenging activity of DPPH radical of Gijlar pepper extract for 30 and 60 min at 65 °C was increased and in red pepper for 60 min at 20 and 35 °C was significantly different (p<0.05) in compare with another groups. As shown in Figure 2, scavenging activity of hydrogen peroxide radical (A) of extracts was different in various temperatures. This ability was the highest in Gijlar pepper at 20 °C and 35 °C for 30 min and in red pepper at 35 °C. Chain-breaking activity (B) of red pepper extract for 60 min at 35 °C was increased and at 50 °C was decreased. Chain-breaking activity of Gijlar pepper extract at all temperature conditions was low and in red pepper for 60 min at 35 °C was the highest and significantly different (p<0.05) in compare with another groups. FRAP (C) of red pepper extract was significantly different (p<0.05) in compare with Gijlar pepper. 

## Discussion

Processing techniques involving extraction solvent, pH, light, and heat can markedly influence the levels and efficacy of bioactive compounds of dietary supplements such as polyphenolic compounds. Polyphenolic constituents of botanicals are unstable compounds and their degradative reactions occurrs throughout the stages of formulation process of a dietary supplement. Botanical extracts and beverages involve heating, which may have an impact on bioactivity. Beverages in general require pasteurization known to affect the activity of some polyphenols. Moreover, antioxidants properties of phenolics make them sensitive to oxidation (Akowuah et al., 2009 ▶). 

In most studies on the effects of heat treatment on the total phenolic content, the results are contradicting. Some researchers reported an increase in the phenolic content whilst others observed a decrease (Chipurura et al., 2010 ▶). Lima et al. (2009) ▶ observed a dramatic loss of phenolic content in edible vegetables as a result of thermal treatment. Decreasing temperature of processing was also found to preserve 80-100% of phenolic content in some vegetables (Roy et al., 2007 ▶). Lopez et al. (2010) ▶ observed that an increase in drying temperature had an important effect on the total phenolic content of blueberry varieties compared with the fresh sample. Long drying times associated with low process temperatures (e.g., 50, 60, and 70 °C) contribute to diminish the protective effect against oxidative damage to cells. Furthermore, a significant increase in polyphenols concentration observed at high temperature (e.g., 90 °C) was probably due to generation of different antioxidant compounds with a varying degree of antioxidant activity.

Chen et al. (2011) ▶ observed that when the citrus fruit (*Citrus sinensis* (L.) Osbeck) peels were dried at 50 and 60 °C, the total phenolic contents were significantly lower than those of fresh peels. However, the phenolic content gradually increased as drying temperature increased. The highest total phenolic content was in the peel dried at 100 °C. Its content was increased around two-fold compared with that of the fresh peel. Jeong et al. (2004) ▶ mentioned that the total phenolic contents in 70% ethanol extracts of *Citrus unshiu* peel (CP) increased with heating temperature, and several low molecular weight phenolic compounds might form in a CP heated at 150 °C for 30 min. Que et al. (2008) ▶ indicated that the formation of phenolic substances in pumpkin (*Cucurbita moschata* Duch.) occurred during drying at 70 °C and mentioned that the formation of phenolic compounds might be due to the availability of precursors of phenolic molecules by non-enzymatic inter conversion between phenolic molecules.

Chen et al. (2011) ▶ observed that the total flavonoid contents of orange (*Citrus sinensis* (L.) Osbeck) peel extracts decreased with lower heating temperature (<80 °C) and increased with higher heating temperature (>90 °C). Ho and Lin (2008) indicated that the flavonoid contents increased with heating time, but the total flavanone glycoside content of the 150 °C heated Huyou peel was lower than that of the 120 °C (Xu et al., 2007). Moreover, the total flavanone glycoside content of the Huyou peel treated at 120 °C for 90 min was lower than that of the peel treated for 60 min. Therefore, a higher temperature (>100 °C) treatment might destroy the flavonoid compounds of citrus peel (Chen et al., 2011 ▶). 

Aoyama and Yamamoto (2007) ▶ studied the antioxidant activity and flavonoid content of Welsh onion (*Allium fistulosum*) and the effect of thermal treatment. The percentages of total flavonoid in boiling water of all of the four vegetables examined were less than 5% of the original raw vegetables. The percentages of total flavonoid in boiling water in yellow and red onions slightly increased during the boiling time from 15 to 60 min.The DPPH radical is long-lived organic nitrogen radical and has a deep purple colour. It is commercially available and does not have to be generated before assay. In this assay, the purple chromogen radical is reduced by antioxidant/reducing compounds to the corresponding pale yellow hydrazine. The reducing ability of antioxidants towards DPPH can be evaluated by electron spin resonance or by monitoring the absorbance decrease at 515-528 nm until the absorbance remains stable in organic media. This widely used method was first reported by Brand-Williams et al. (1995) ▶. 

The radical scavenging activity of blueberry varieties was investigated based on air-drying temperature. Dehydration at high temperatures (e.g., 80 and 90 °C) shows higher antioxidant activity rather than at low temperatures (e.g., 50, 60, and 70 °C) (Lopez et al., 2010 ▶). This behaviour could be related to drying process at low temperatures which implies long drying times may cause a decrease of antioxidant activity (Garau et al., 2007 ▶). Chen et al. (2011) ▶ observed that the highest DPPH radicals scavenging effect was seen in the citrus fruit (*Citrus sinensis* (L.) Osbeck) peel extracts from the 100 °C dried treatment. The effects of fresh (control) and lower temperature treatments (50, 60, and 70 °C) increased slower than the higher temperature treatments (80, 90, and 100 °C). This result indicates that a higher drying temperature treatment can improve the DPPH radical scavenging effect of orange peel. Jeong et al. (2004) ▶ and Ho and Lin (2008) ▶ showed that the DPPH radical scavenging effect of extracts from citrus peels increased with heating time. 

The radical scavenging activity of red pepper (*C. annuum* var. Hungarian) was investigated based on air-drying temperature. Dehydration at high temperatures (i.e., 80 and 90 °C) shows higher antioxidant activity rather than at low temperatures (i.e., 50, 60, and 70 °C) (Vega-Galvez et al., 2009 ▶). This behaviour could be related to drying process at low temperatures, which implies long drying times may promote a decrease of antioxidant capacity (Garau et al., 2007 ▶). Furthermore, generation and accumulation of Maillard-derived melanoidins having a varying degree of antioxidant activity could also enhance antioxidant properties at high temperatures (i.e., 80 and 90 °C) (Miranda et al., 2009 ▶; Que et al., 2008 ▶).

Hydrogen peroxide exhibits weak activity in initiating lipid peroxidation; however, its potential to produce highly reactive oxygen species (ROS), such as hydroxyl radical through Fenton reaction, is very high. Hydrogen peroxide is poorly reactive in aqueous solutions at physiological concentrations and is toxic to cells at 10-100 µ levels, and can cross biological membranes rapidly to form cytotoxic hydroxyl radicals (Siriwardhana and Shahidi, 2002 ▶). The hydrogen peroxide-scavenging activity of almond hulls and shells (*Amygdalus communis* L.) methanolic extracts were phenol content dependent and in genotypes with high phenolic content, especially hull extracts, most of the hydrogen peroxide was scavenged. The rates of hydrogen peroxide scavenging of hulls and shells vary among genotypes. Hydrogen peroxide-scavenging activity of the hull was higher than of the shell in each genotype. Thus, hydrogen peroxide-scavenging activity of almond hulls and shells extracts, especially hull extracts would contribute to their inhibition of lipid peroxidation and thereby protect cells from oxidative damage (Jahanban Sfahlan et al., 2009 ▶).

(Madrau et al., 2009 ▶) showed that the antioxidant capacity and chain-breaking activity increased significantly in Cafona apricot as a consequence of increasing drying temperature, while the values did not change in the Pelese apricot. These data are confirmed by changes in redox potential values, which decreased from 382.5 mV on fresh fruit to 340 and 319 mV for Cafona apricot dried respectively at 55 and 75 °C, indicating an increase in the reducing properties of fruits. The chain-breaking activity of the Allium (*Allium cepa* L., *Allium sativum* L.) extracts was interesting to note that heating caused an increase in chain-breaking activity which was very high in case of ascorbic acid. Comparatively with Allium extracts, initial chain breaking of ascorbic acid was from 12- to 208-fold higher. After three hours at 90 ºC, k value was from 15 to 298 fold higher (Benkeblia, 2005 ▶).

The FRAP assay is based on the ability of phenolics to reduce Fe^3+ ^to Fe^2+^. When this occurs in the presence of TPTZ, the reduction is accompanied by the formation of a colored complex with Fe^2+^ (Roginsky and Lissi, 2005 ▶). The reducing capacity of compound may serve as a significant indicator of its potential antioxidant activity (Meir et al., 1995 ▶). The reducing power of Maillard reaction products derived from L-ascorbic acid and L-methionine model system, as measured by A_700_, reached a maximum at 200 min of heating time. A slight decrease was observed with increasing heating time up to 500 min (Deng et al., 2011 ▶). An increase in the antioxidant activities determined by FRAP assays in the treated vegetables was observed in comparison with the raw ones, which were explained by the conversion of antioxidants to higher amounts of antioxidant chemical species (Miglio et al., 2008 ▶).

This study suggested that an appropriate temperature maintained a high antioxidant activity of phenolic compound, which could be due to the combined effect of nonenzymatic reaction and phenolic compound stability (Reyes and Cisneros-Zevallos, 2007 ▶).

In this study, it was demonstrated that various temperatures influenced the antioxidant activity of sweet bell pepper phenolic extracts. The extracts from sweet bell pepper possess antioxidant and antiradical activity, which could vary in different varieties, temperatures values, and this phenolic extract, may be helpful in preventing or slowing the progress of various oxidative stress-related diseases. The results were different depending on the temperature and varieties.
